# Localized Lymph Node Light Chain Amyloidosis

**DOI:** 10.1155/2015/816565

**Published:** 2015-04-02

**Authors:** Binod Dhakal, Alexandra M. Harrington, Michael E. Stadler, Anita D'Souza

**Affiliations:** ^1^Division of Hematology & Oncology, Medical College of Wisconsin, 9200 W. Wisconsin Avenue, Milwaukee, WI 53226, USA; ^2^Department of Pathology, Medical College of Wisconsin, 9200 W. Wisconsin Avenue, Milwaukee, WI 53226, USA; ^3^Department of Otolaryngology and Communication Sciences, Medical College of Wisconsin, 9200 W. Wisconsin Avenue, Milwaukee, WI 53226, USA

## Abstract

Immunoglobulin-derived light chain amyloidosis can occasionally be associated with localized disease. We present a patient with localized lymph node light chain amyloidosis without an underlying monoclonal protein or lymphoproliferative disorder and review the literature of lymph node amyloidosis discussing work-up and risk factors for systemic progression.

## 1. Introduction

Immunoglobulin-derived amyloidosis includes a group of diseases associated with the deposition of misfolded insoluble immunoglobulin chains, commonly light (AL), rarely heavy (AH) chains [1]. These diseases are associated with hematologic malignancies resulting in an overproduction of immunoglobulin chains including the plasma cell disorders (multiple myeloma, primary systemic amyloidosis, and plasmacytoma) and lymphoproliferative disorders (chronic lymphocytic leukemia, lymphoplasmacytic lymphoma including Waldenstrom macroglobulinemia, and marginal zone lymphoma) [2]. Additionally, the spectrum of immunoglobulin-derived amyloidosis may range between systemic disease characterized by widespread amyloid deposition in organs distal to the site of production, that is, paraneoplastic, and a more localized form of disease where the amyloid remains at the site of production, that is, peritumoral [3]. Localized forms of immunoglobulin-derived amyloidosis include lower urinary tract amyloid [4], pulmonary (tracheobronchial and nodular pulmonary) amyloid [5, 6], head and neck (oropharyngeal, laryngeal) amyloidosis [7, 8], and gastrointestinal amyloidosis [9, 10]. In 2–5% of AL amyloidosis, an underlying B cell lymphoproliferative disorder may be found [11, 12]. The lymphoproliferative disorders can range between lymphoplasmacytic lymphoma, chronic lymphocytic leukemia, and marginal zone lymphoma [13–16]. Localized primary amyloidosis is associated with single organ involvement and has low level of monoclonal protein. Truly localized lymph node amyloidosis without a monoclonal protein component is very rare. Herein, we present a patient with localized amyloidosis of the supraclavicular lymph nodes with no evidence of a monoclonal protein component. We review the literature to assess risk factors for systemic disease and prognosis.

## 2. Case Presentation

**Figure 1:**
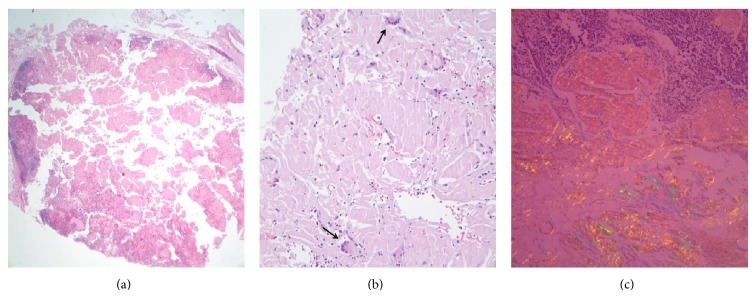
(a) Lymph node replaced by amorphous, eosinophilic, extracellular material (amyloid), (b) high power magnification of the amyloid with few admixed multinucleated giant cells (arrowed), and (c) Congo red stain demonstrating apple green birefringence, diagnostic of amyloid.

**Figure 2:**
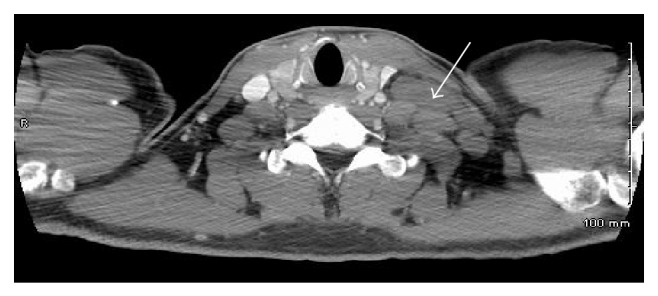
CT scan of the neck with left supraclavicular lymphadenopathy (arrow).

A 46-year-old Caucasian male presented to his primary care physician with a painless neck mass. A computerized tomography (CT) scan of the neck and chest was obtained which revealed a left supraclavicular soft tissue lymph node mass. A fine needle aspiration biopsy demonstrated amyloid deposits suggesting amyloidoma. The patient was observed for a year when he noticed a slight increase in the size of the mass concurrent with an upper respiratory illness. He was referred to a hematologist at our center. The amyloidoma was subject to mass spectrometry-based proteomic analysis, which revealed this to be of AL (kappa) subtype. There was no evidence of a monoclonal protein on serum and 24-hour urine protein electrophoresis, immunofixation electrophoresis, and serum-free light chain analysis. He underwent a bone marrow aspiration/biopsy and a fat pad aspirate, both of which were unremarkable and showed no evidence of amyloidosis by Congo red staining. There was no evidence of solid organ amyloidosis based on clinical and focused laboratory and imaging studies. A CT scan of his chest, abdomen, and pelvis did not reveal any evidence of a lymphoproliferative disorder. Comparison of his previous imaging demonstrated stable 3.5 cm amyloidoma in the left supraclavicular fossa (Figure 2). Because of his local symptoms, he was referred to a head and neck surgeon and underwent a complete excision of this mass which histologically showed predominant fibroadipose tissue with extensive amyloid deposition (Figure 1). No evidence of an underlying clonal B cell or plasma cell population was found. No specific therapy was indicated or offered after the resection of the mass. The patient continues to remain asymptomatic with no evidence of lymphadenopathy, mass lesion, or any other systemic evidence of amyloidosis.

## 3. Discussion

Localized immunoglobulin-derived amyloidosis is a well-reported entity and can be seen in a wide variety of organs including the lower urinary and aerodigestive tracts. Lymph node amyloidosis, however, appears to be a distinct entity and can present with localized and/or systemic involvement. When seen in systemic AL amyloidosis, amyloid lymphadenopathy may occur with a frequency ranging from 17 to 37% [17]. Pathophysiologically, lymph node amyloidosis has been associated with an underlying clonal lymphoproliferative disorder such as lymphoplasmacytic lymphoma, marginal zone lymphoma, and chronic lymphocytic leukemia [3, 11, 15, 16] and may be associated with an IgM monoclonal paraproteinemia [12–14]. Additionally, when localized, lymph node amyloidosis has been reported to have an AH component (AH or AH/AL) more often [2]. When a diagnosis of lymph node amyloidosis is made, it is important to distinguish between localized and systemic forms as patients with a localized presentation are thought to have a better prognosis [2, 3]. Consequently, patients with localized amyloidosis do not require treatment with systemic chemotherapy or stem cell transplantation. In a large series of lymph node amyloidosis cases described from a single institution, localized lymph node involvement by amyloid was found to be associated with either a peritumoral distribution defined as amyloid restricted to sites of detectable lymphoma or localized defined as lymph node amyloidosis without lymphoma or a circulating monoclonal protein [2]. Truly localized lymph node amyloidosis was only seen in 2 patients, both with AH amyloid with a speculation that heavy chains by virtue of their size may be more pathogenic to the local site of production and less likely to affect the distant organs [2]. Our patient would be considered a localized lymph node amyloidosis by that definition, albeit with the unusual finding of only AL subtype. In a single institution of 20 patients, 1 patient had lymph node amyloidosis [18]. Similarly in another series of 9 patients with AH amyloidosis, only 2 patients had localized disease [19]. A recent series of amyloid lymphadenopathy cases from the Boston University reported that the majority of patients who present with isolated lymphadenopathy eventually progress to develop other organ diseases and therefore need a thorough evaluation for systemic organ involvement and regular monitoring [20]. In their series [20], out of 3008 patients with all forms of amyloidosis, a total of 47 patients (2%) presented with lymphadenopathy as the presenting manifestation of amyloidosis. Of those, only 14 patients had isolated lymphadenopathy without involvement of other organs, but 10 of those eventually developed systemic disease requiring systemic chemotherapy. The median overall survival was much higher in this group of patients than the entire cohort. Four patients were observed with no adverse outcomes or signs of vital organ involvement or plasma cell dyscrasia. It is interesting to note that majority of those progressed into either plasma cell dyscrasia or lymphoma and very few had local progression [20]. Thus, based on published literature, true localized amyloid lymphadenopathy, as seen in our patient, appears to be very rare whereas systemic amyloidosis with lymphadenopathy appears more likely. Certainly, once a diagnosis of amyloid lymphadenopathy is made, a systematic search for distant organ involvement is imperative.

The prognosis of localized immunoglobulin-derived amyloidosis is reported to be good and often involves expectant management or localized therapies if symptomatic, without the need for chemotherapies, in contrast to systemic AL amyloidosis. This appears to be the case for truly localized amyloid lymphadenopathy as well [2, 3, 10]. Localized amyloidosis associated with a lymphoproliferative disorder in other locations also appears to have a good outcome without development of systemic amyloid disease [16]. While it is unclear why some amyloidosis remains localized but disseminates systemically, there is reason to believe that localized immunoglobulin-derived amyloidosis may have different composition including more AH or mixed AH/AL deposition compared with systemic forms which are predominantly AL [2, 6, 19, 21]. There are no known risk factors that could predict the progression into systemic disease but different case series have shown that those without a measurable peripheral monoclonal protein were less likely to develop systemic disease [2, 17, 18]. Additionally, the presence of heavy chain in the amyloid deposits may also suggest a localized form of amyloidosis [2, 6].

In conclusion, we present a patient with localized lymph node AL amyloidosis without evidence of underlying monoclonal protein or lymphoproliferative disorder. This case is remarkable from published literature of localized amyloidosis by absence of underlying paraproteinemia, lymphoma, or a heavy chain component in the amyloid composition. While it is important to systematically evaluate these patients for evidence of systemic amyloidosis, it is also equally important to treat them appropriately, with observation or resection if there is no evidence of systemic disease. Similar to other localized AL amyloidosis cases, the course may be indolent with an overall excellent prognosis with minimal intervention.
